# Adjuvant and neoadjuvant therapy with or without CDK4/6 inhibitors in HR+/HER2- early breast cancer: a systematic review and meta-analysis

**DOI:** 10.3389/fphar.2024.1438288

**Published:** 2024-09-12

**Authors:** Zhihao Zhang, Xin Zhao, Jie Chen

**Affiliations:** Breast Center, Department of General Surgery, West China Hospital, Sichuan University, Chengdu, Sichuan, China

**Keywords:** CDK4/6 inhibitors, HR-positive, adjuvant, breast cancer, endocrine therapy

## Abstract

**Background:**

The combination of cyclin-dependent kinases 4/6 (CDK4/6) inhibitors and endocrine therapy is the standard treatment for patients with hormone receptor-positive (HR+)/HER2-negative (HER2-) advanced breast cancer. However, the role of CDK4/6 inhibitors in early breast cancer remains controversial.

**Methods:**

This study aimed to evaluate the efficacy and safety of CDK4/6 inhibitors combined with endocrine therapy versus endocrine therapy alone in patients with HR+, HER2- early breast cancer. A systematic review of Cochrane, PubMed and EMBASE databases was conducted. The efficacy endpoints of adjuvant therapy were invasive disease-free survival (IDFS), overall survival (OS) and distant relapse-free survival (DRFS). The efficacy endpoint included complete cell cycle arrest (CCCA) and complete pathologic response (PCR) with neoadjuvant therapy. Grade 3/4 adverse events (AEs) were assessed as safety outcomes.

**Results:**

Eight randomized controlled trials (RCTs) were included in the study. CDK4/6 inhibitors combined with endocrine therapy showed a significant improvement in IDFS (hazard ratio (HR) = 0.81, 95% confidence interval (CI) = 0.68–0.97, *P* = 0.024), but not DRFS (HR = 0.84, 95% CI = 0.56–1.29, *P* = 0.106) or OS (HR = 0.96, 95% CI = 0.77–1.19, *P* = 0.692) in adjuvant therapy. In the neoadjuvant therapy setting, CDK4/6 inhibitors improved CCCA compared with the control group (RR = 2.08, 95% CI = 1.33–3.26, *P* = 0.001). The risk of 3/4 grade AEs increased significantly with the addition of CDK4/6 inhibitors to endocrine therapy.

**Conclusion:**

The addition of CDK4/6 inhibitors in HR+/HER2- early breast cancer patients significantly improved IDFS in adjuvant therapy and CCCA in neoadjuvant. However, CDK4/6 inhibitors also showed significant toxicities during therapy.

**Systematic Review Registration:**

Identifier CRD42024530704.

## Introduction

Breast cancer has emerged as the most prevalent malignant neoplasm worldwide, with over 90% of breast cancer patients diagnosed with early-stage disease, among which the most common subtype is hormone receptor (HR)-positive (Cardoso et al., 2018). Treatment strategies for such patients vary based on the risk of recurrence and include combinations of surgery, chemotherapy, radiotherapy, and endocrine therapy (Harbeck and Gnant, 2017). Although standard treatments for HR-positive breast cancer have notably improved over the years, some patients do not respond to endocrine therapy due to intrinsic or acquired resistance (Burstein et al., 2021; Johnston et al., 2020). Therefore, numerous pharmaceuticals are under development to address the challenge of endocrine resistance (Burstein, 2020).

Cyclin-dependent kinases (CDKs) are a class of serine/threonine kinases that regulate cell cycle progression (Gomes et al., 2023). Numerous preclinical studies have demonstrated that luminal breast cancer exhibits overactivity in the CDK4/6-cyclin D1 pathway, which provides a strong rationale for the therapeutic efficacy of CDK4/6 inhibitors (O’Sullivan et al., 2023).

Currently, three pharmaceutical agents, namely, abemaciclib, palbociclib, and ribociclib, have obtained approval from both the FDA and the European Medicines Agency (EMA), demonstrating clear benefits in HR-positive/HER2-negative advanced or metastatic breast cancer (Eggersmann et al., 2019). This has also heightened our interest in determining the benefits for patients with HR-positive/HER2-negative early breast cancer. However, previous studies have indicated controversies regarding the efficacy of CDK4/6 inhibitors in combination with endocrine therapy for HR-positive/HER2-negative early breast cancer (Slamon et al., 2024; Gnant et al., 2022; Loibl et al., 2021). NATALEE (Slamon et al., 2024) and MonarchE (Johnston et al., 2023) trails found survival benefits of CDK4/6 inhibitors combined with endocrine therapy in HR-positive early breast cancer, whereas the results of the other two studies (Gnant et al., 2022; Loibl et al., 2021) indicated that CDK4/6 inhibitors did not improve survival outcomes in these patients.

Therefore, this meta-analysis included all available randomized controlled trials (RCTs) aimed at exploring the efficacy and safety of CDK4/6 inhibitors combined with endocrine therapy in the adjuvant or neoadjuvant treatment of patients with HR-positive/HER2-negative early breast cancer.

## Methods

### Study objectives

This study aimed to evaluate the efficacy of CDK4/6 inhibitors in combination with endocrine therapy as adjuvant or neoadjuvant treatment in patients with HR-positive/HER2-negative early breast cancer. The primary efficacy endpoint of adjuvant therapy was invasive disease-free survival (IDFS), with secondary endpoints including overall survival (OS), distant relapse-free survival (DRFS), and grade 3/4 adverse events (AEs). The efficacy endpoints of neoadjuvant therapy were complete cell cycle arrest (CCCA; defined as Ki67 ≤ 2.7%) and pathological complete response (PCR).

### Search strategy

This systematic review and meta-analysis were conducted according to the Preferred Reporting Items for Systematic Reviews and Meta-Analyses (PRISMA) guidelines (Moher et al., 2009) and was registered in the PROSPERO database (ID: CRD42024530704). The systematic search was conducted in three electronic databases (PubMed, Embase, and Cochrane Library) up to April 2024. The terms used in the search strategy were related to “breast cancer” and “cyclin-dependent kinase 4/6 inhibitor.” The detailed search strategy is available in Supplementary Table S1.

### Inclusion and exclusion criteria

The inclusion criteria were as follows: 1) the study was an RCT; 2) the study population comprised patients pathologically diagnosed with HR-positive/HER2-negative early breast cancer; 3) the study included patients treated with CDK4/6 inhibitors in combination with endocrine therapy versus endocrine therapy with or without placebo; 4) the endpoint information of the study included one or more IDFS, DRFS/DDFS, OS, CCCA, PCR and grade 3/4 AEs; and 5) the study was published in English.

The exclusion criteria were as follows: 1) non-RCT studies; 2) single-arm tests; 3) systematic reviews, meta-analyses, case reports, and animal studies; and 4) studies with insufficient information for meta-analysis.

### Data extraction

For studies meeting the inclusion criteria, the following information was independently extracted by two investigators (ZZ and WL): study name, phase, sample size, menopausal status, treatment, CDK4/6 inhibitor duration and endpoints. Any disagreements were resolved by another investigator (JC).

### Assessment of study quality

The Cochrane Collaboration risk-of-bias tool (Higgins et al., 2011) was used to assess the quality of the included RCTs. Literature quality evaluation was conducted by Review Manager, version 5.3. The quality assessment criteria included selection bias, performance bias, detection bias, attrition bias, reporting bias and other biases.

### Statistical analysis

Analyses were conducted using Review Manager software (version 5.3). Hazard ratios (HRs) and 95% CIs were utilized for IDFS, DRFS/DDFS, OS, and subgroup analyses. CCCA, PCR and grade 3/4 AEs were analyzed using risk ratios (RRs). The cutoff for statistical significance was *P* < 0.05. This study’s heterogeneity assessment was conducted using Cochran’s Q and I^2^ tests (Higgins et al., 2003). A value greater than 50% for I^2^ and *P* < 0.1 for Cochran’s Q indicated the presence of heterogeneity, and a random-effects model was employed for analysis (Zhang et al., 2024). Conversely, a fixed-effects model was utilized. Egger’s test was also used to assess potential publication bias (Egger et al., 1997).

## Results

### Characteristics of the included studies

**FIGURE 1:**
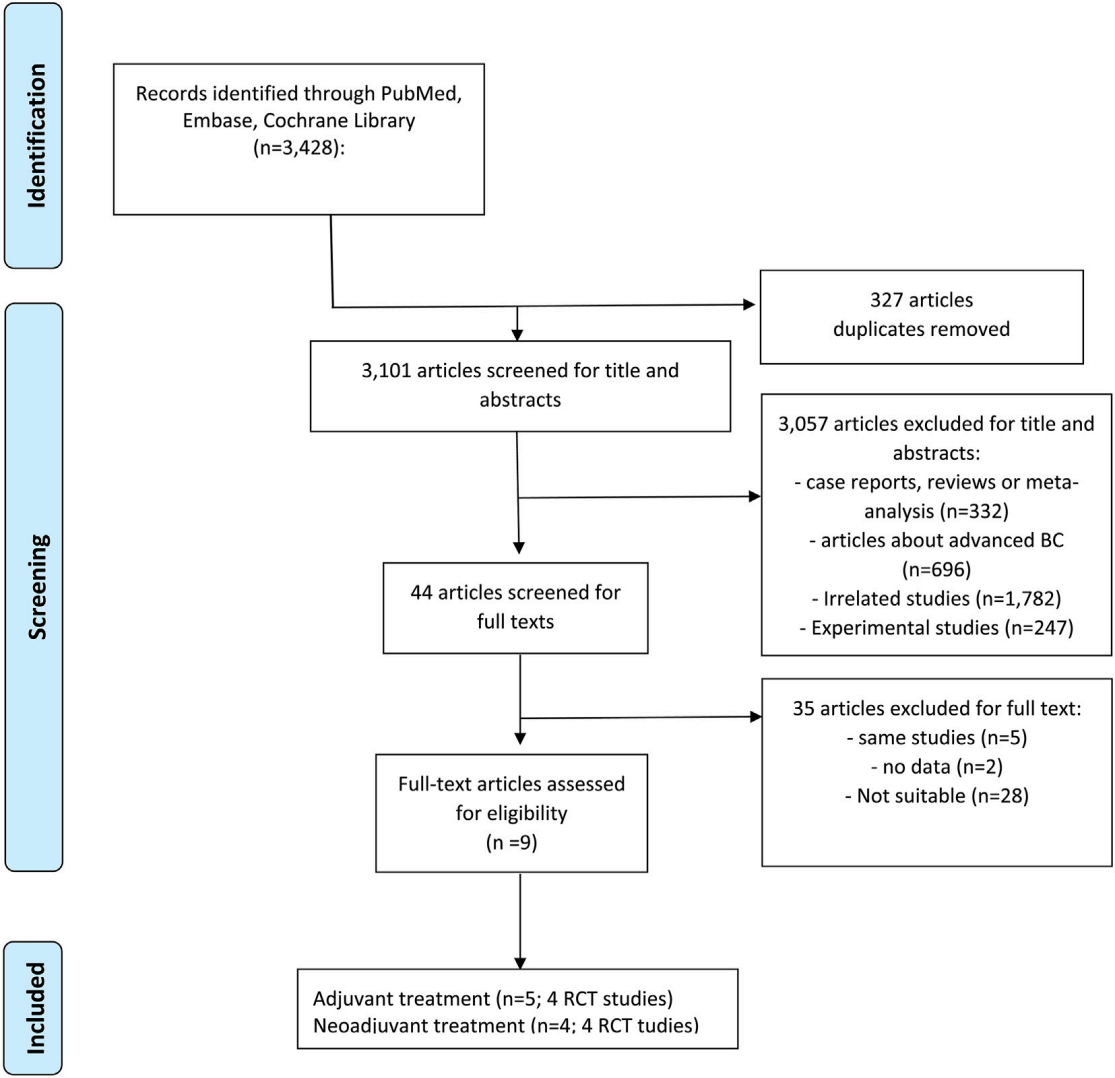
PRISMA flowchart. PRISMA flowchart of retrieved studies. (Abbreviations: BC, breast cancer; RCT, randomized controlled trials).

In total, 3,428 records were initially identified from three electronic databases, 44 studies remained after duplicate removal and title and abstract were screened, and 35 were removed for the following reasons: 5 were the same or subgroups of RCT trials, 2 had insufficient information, and 28 were unrelated studies (Figure 1). Finally, 9 studies (8 RCT trials) that met our inclusion criteria were included (Slamon et al., 2024; Gnant et al., 2022; Loibl et al., 2021; Johnston et al., 2023; Rastogi et al., 2024; Alsaleh et al., 2023; Hurvitz et al., 2020; Johnston et al., 2019; Khan et al., 2020).

Detailed information on the included studies is shown in Table 1. Four studies (Slamon et al., 2024; Gnant et al., 2022; Loibl et al., 2021; Johnston et al., 2023), including 17,749 patients, evaluated CDK4/6 inhibitors in the adjuvant setting, and another four studies (Alsaleh et al., 2023; Hurvitz et al., 2020; Johnston et al., 2019), including 804 patients, evaluated CDK4/6 inhibitors in the neoadjuvant setting. It is worth noting that the MonarchE trial recently provided updated efficacy-related data but did not include safety-related data. Therefore, we included two published articles for this trial (Johnston et al., 2023; Rastogi et al., 2024).

**TABLE 1:** Characteristics of the studies included in the meta-analysis.

Study	Phase	Treatment	NCT	Median age (years)	N (experimental/control)	CDK4/6 inhibitor duration	Median follow-up (months)	Endpoints
Adjuvant therapy								
NATALEE (Slamon et al., 2024)	III	Ribociclib + AI vs. AI	NCT03701334	52	2,549/2,552	3 years	34	IDFS, OS, AEs
PALLAS (Gnant et al., 2022)	III	Palbociclib + AI/tamoxifen vs. AI/tamoxifen	NCT02513394	52	2,884/2,877	2 years	31	IDFS, DRFS, OS, AEs
MonarchE (Rastogi et al., 2024)	III	Abemaciclib + AI/tamoxifen vs. AI/tamoxifen	NCT03155997	51	2,808/2,829	2 years	54	IDFS, OS, AEs
PENELOPE-B (Loibl et al., 2021)	III	Palbociclib + AI/tamoxifen vs. Placebo + AI/tamoxifen	NCT01864746	49	631/619	1 year	42.8	IDFS, DRFS, OS, AEs
Neoadjuvant therapy								
SAFIA (Alsaleh et al., 2023)	III	Fulvestrant + palbociclib versus Fulvestrant + placebo	NCT03447132	49	114/115	16 weeks	NA	PCR
FELINE (Khan et al., 2020)	II	Ribociclib + Letrozole vs. Placebo + Letrozole	NCT02712723	NA	82/38	14 weeks	Baseline, day 14 cycle 1 (D14C1), and surgery	CCCA
PALLET (Johnston et al., 2019)	II	Palbociclib + Letrozole vs. Letrozole	NCT02296801	65.1	204/103	16 weeks	Baseline, 2 weeks and 14 weeks	CCCA, PCR
neoMONARCH (Hurvitz et al., 2020)	II	Abemaciclib + Anastrozole vs. Anastrozole	NCT02441946	64	74/74	26 weeks	Baseline, 2 weeks, and the end of treatment (16 weeks)	CCCA

NCT, national clinical trial number; AEs, adverse events; AI, aromatase inhibitor; CDK, cyclin dependent kinase; DRFS, distant relapse-free survival; IDFS, invasive disease-free survival; OS, overall survival; CCCA, complete cell cycle arrest.

The study quality assessment is shown in Supplementary Figure S1. The open-label design of the four studies (NATALEE, PALLAS, NeoMonarchE, and MonarchE) led to a high risk of performance bias, as it could result in outcome assessments being influenced by knowledge of the intervention. However, a high risk of bias was not observed for the remaining biases.

### Adjuvant therapy

**FIGURE 2:**
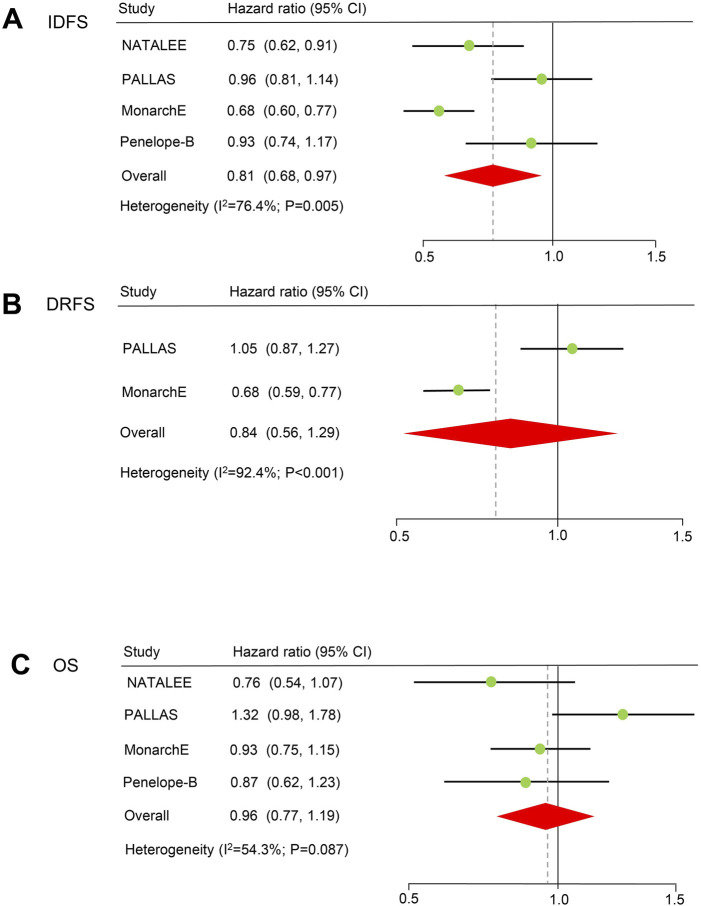
Forest plots of pooled hazard ratios for adjuvant therapy. **(A)** IDFS after adjuvant therapy. **(B)** DRFS after adjuvant therapy. **(C)** OS of patients receiving adjuvant therapy. IDFS: invasive disease-free survival. OS: overall survival. DRFS: distant relapse-free survival.

IDFS: Four RCTs reported the IDFS of patients treated with CDK4/6 inhibitors and the recruitment criteria were shown in Supplementary Table S2, and the results showed a significant improvement in IDFS (HR = 0.81, 59% CI = 0.68–0.97; *P* = 0.024; Figure 2). A random-effects model was used because obvious heterogeneity (I^2^ = 76.4%, *P* = 0.005) existed. According to the majority of subgroup analyses (Figure 2), CDK4/6 inhibitors significantly improved survival according to menopausal status, nodes status, stage and Asian (*P* < 0.05). Although there was no statistical significance in the remaining subgroups, there was still a trend toward prolonged IDFS (HR < 1). Notably, heterogeneity disappeared in the menopausal status and tumor stage subgroups (Table 2). In the sensitivity analysis, there was no significant difference when excluding the NATALEE study, but there was still a trend toward improvement in the efficacy of CDK4/6 inhibitors (Supplementary Table S3). Egger’s test did not detect potential publication bias (*P* = 0.285).

**TABLE 2:** Subgroup analysis of IDFS.

Subgroup	Studies	Hazard ratio (95% CI)	P	I^2^ (%)
Age				
≤50	2	0.98 (0.83, 1.23)	0.923	0
>50	2	0.90 (0.74, 1.09)	0.281	0
Menopausal status				
Premenopausal	2	0.63 (0.54, 0.75)	<0.001	0
Postmenopausal	2	0.76 (0.66, 0.87)	<0.001	0
T				
T1-2	2	0.79 (0.57, 1.11)	0.176	60.7
T3	2	0.85 (0.54, 1.32)	0.462	81.0
N				
N0-1	3	0.82 (0.69, 0.98)	0.026	0
N2-3	2	0.78 (0.60, 1.02)	0.067	76.1
Stage				
I	2	0.76 (0.61, 0.95)	0.018	0
II	2	0.68 (0.60, 0.77)	<0.001	0
Grade				
1-2	2	0.76 (0.58, 1.01)	0.056	72.9
3	2	0.84 (0.64, 1.10)	0.196	62.8
Region				
Asian	2	0.65 (0.49, 0.85)	0.002	0
Non-Asian	2	0.80 (0.59, 1.08)	0.144	76.7

IDFS, invasive disease-free survival; CI, confidence interval.

DRFS Two RCTs (PALLAS and PENELOPE-B) reported the DRFS of patients treated with CDK4/6 inhibitors, and the results indicated that CDK4/6 inhibitors may prolong DRFS, but the difference was not statistically significant (HR = 0.84, 59% CI = 0.56–1.29; *P* = 0.422; Figure 2). A random-effects model was used because obvious heterogeneity (I^2^ = 92.4%, *P* < 0.001) existed. Egger’s test could not be conducted because only two studies were included.

OS Four RCTs reported the OS of patients treated with CDK4/6 inhibitors, and there was no statistically significant difference in terms of OS (HR = 0.96, 59% CI = 0.77–1.19, *P* = 0.692). A random-effects model was used because heterogeneity (I^2^ = 54.3%, *P* = 0.087) existed. Egger’s test did not detect potential publication bias (*P* = 0.879).

AEs: Four RCTs reported the adverse effects of CDK4/6 inhibitors. The results showed a significant increase in the incidence of any grade 3 or 4 AEs (RR = 3.70, 59% CI = 2.81–4.88, *P* < 0.001) in the CDK4/6 inhibitor combined with endocrine therapy group compared to the group receiving only endocrine therapy, and obvious heterogeneity was detected (I^2^ = 95.8%, *P* < 0.001). In particular, CDK4/6 inhibitors were significantly associated with grade ≥3 AEs, such as neutropenia, anemia, leukopenia, thrombocytopenia, alanine aminotransferase (ALT), aspartate aminotransferase (AST), nausea, headache, and back pain (Table 3).

**TABLE 3:** Pooled risk ratio of adverse events in adjuvant therapy.

AEs (grade 3-4)	Studies	Risk ratio (95% CI)	P	I^2^ (%)
Any	3	3.70 (2.81, 4.88)	<0.001	95.8
Neutropenia	4	60.3 (26.0, 140.1)	<0.001	71.3
Anemia	3	4.38 (2.59, 7.41)	<0.001	0
Leukopenia	3	84.73 (22.16, 323.9)	<0.001	85.4
Thrombocytopenia	2	6.71 (3.00, 15.03)	<0.001	37.1
ALT	3	3.99 (1.31, 12.14)	0.015	87.3
AST	3	5.28 (2.43, 11.51)	<0.001	62.0
Lymphoedema	2	4.54 (0.98, 21.02)	0.053	0
Arthralgia	4	0.64 (0.40, 1.02)	0.059	51.6
Nausea	4	2.82 (1.28, 6.21)	<0.001	15.6
Headache	4	1.54 (0.84, 2.70)	0.175	0
Fatigue	4	5.49 (2.17, 13.88)	<0.001	78.1
Hot flush	4	0.88 (0.47, 1.65)	0.692	3.5
Back pain	2	3.53 (1.16, 10.73)	0.026	0

AEs, adverse events; ALT, alanine aminotransferase; AST, aspartate aminotransferase; CI, confidence interval.

### Neoadjuvant therapy

**FIGURE 3:**
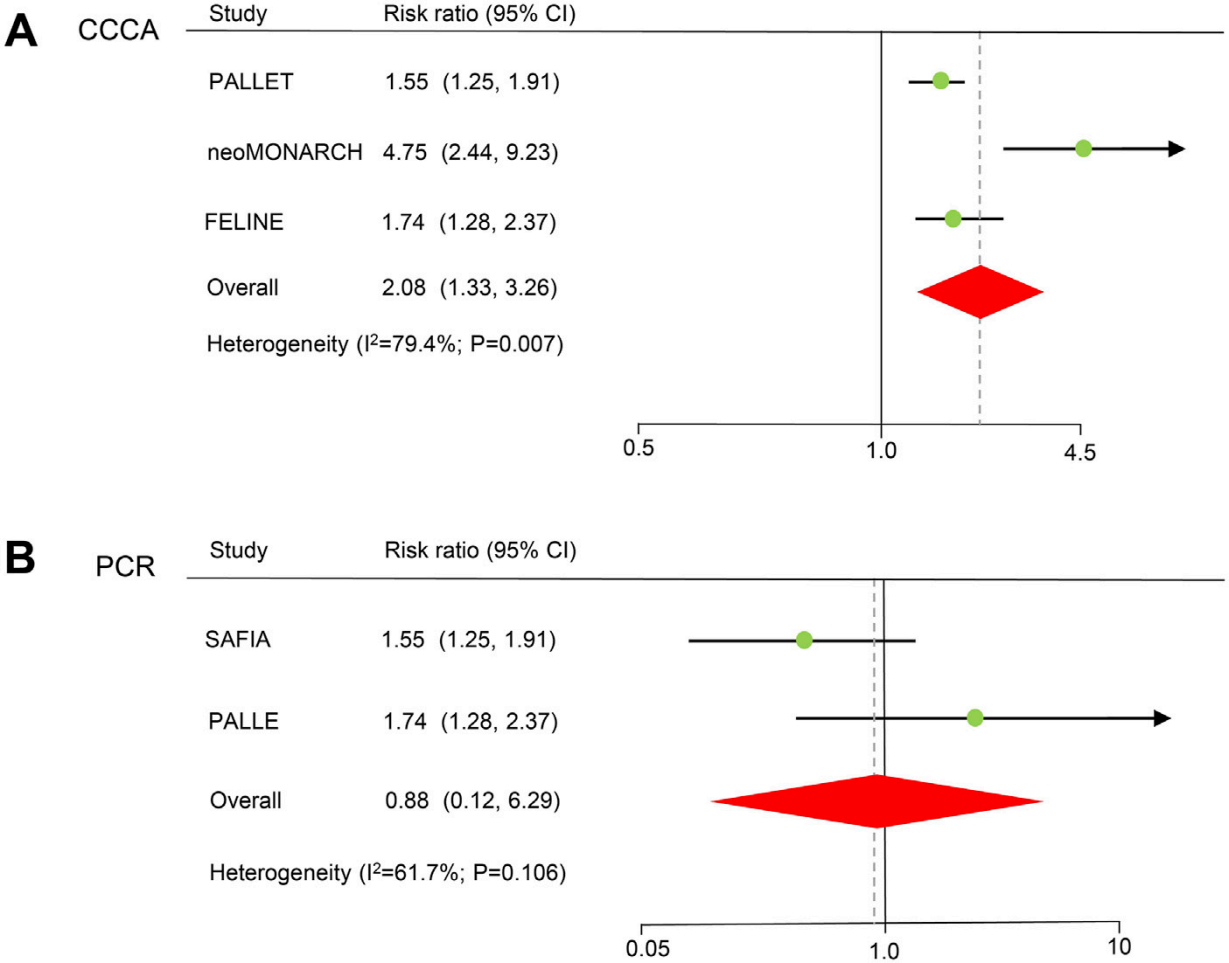
Forest plots of pooled hazard ratios for neoadjuvant therapy. **(A)** CCCA after neoadjuvant therapy. **(B)** PCR of neoadjuvant therapy. CCCA: complete cell cycle arrest. PCR: pathological complete response.

Three neoadjuvant RCT trials reported the CCCA of patients treated with CDK4/6 inhibitors (Figure 3). The results showed a significant improvement in the CCCA (RR = 2.08, 59% CI = 1.33–3.27, *P* < 0.001). A random-effects model was used because obvious heterogeneity (I^2^ = 79.4%, *P* = 0.007) existed. In the sensitivity analysis, there was no significant difference when excluding the FELINE study, but a trend toward improving CCCA still existed (Supplementary Table S4). Egger’s test did not detect potential publication bias (*P* = 0.128).

PCR information for CDK4/6 inhibitors in two neoadjuvant randomized controlled trials. There was no statistically significant difference in terms of PCR (HR = 0.88, 59% CI = 0.12–6.29, *P* = 0.899). A random-effects model was used because heterogeneity (I^2^ = 61.7%, *P* = 0.106) existed.

## Discussion

CDK4/6 inhibitors have yielded definitive results in HR-positive/HER2-negative advanced breast cancer (Kalinsky et al., 2023; Goetz et al., 2017; Lu et al., 2022; Piezzo et al., 2020), as confirmed by previous meta-analyses (Braal et al., 2021; Li et al., 2020a; Li et al., 2020b; Giuliano et al., 2019). There has been increased interest in CDK4/6 inhibitors as potential therapeutic approaches for neoadjuvant and adjuvant therapy for early breast cancer (Haslam et al., 2024). Therefore, this study investigated the efficacy and safety of CDK4/6 inhibitors in the adjuvant and neoadjuvant treatment of HR-positive/HER2-negative early breast cancer. We included eight RCTs for both adjuvant (NATALEE, PALLAS, MonarchE, and Penelope-B) and neoadjuvant (SAFIA, PALLET, NeoMONARCH, and FELINE) therapies.

In our analysis, we revealed a notable benefit in terms of IDFS when comparing the combination of CDK4/6 inhibitors with endocrine therapy to endocrine therapy alone. However, no discernible benefit was observed in terms of DRFS or OS. This finding was inconsistent with the conclusions drawn from previous meta-analyses (Agostinetto et al., 2021), which indicated that CDK4/6 inhibitors confer no benefit on IDFS, DRFS, or OS in HR-positive/HER2-negative early breast cancer patients. The primary factor contributing to this discrepancy is that previous meta-analyses included three RCTs, whereas we additionally included a recently published RCT. Compared to previously published meta-analyses (Agostinetto et al., 2021), our meta-analysis encompasses more comprehensive and complete survival data, references, and subgroup analyses, as well as a larger sample size.

Notably, the PALLAS (Gnant et al., 2022) and Penelope-B (Loibl et al., 2021) trials revealed no benefit in HR+/HER2- early-stage breast cancer, but the NATALEE (Slamon et al., 2024) and MonarchE (Johnston et al., 2023) trials reported divergent findings. Several interpretations have been proposed regarding these discrepancies. First, the MONARCH-E trial exclusively enrolled high-risk patients, suggesting that the favorable outcomes observed with CDK4/6 inhibitors may merely reflect patients with higher-risk diseases (Johnston et al., 2023). However, subgroup analyses of the PALLAS trial data did not demonstrate increased benefits for high-risk patients (Gnant et al., 2022; Morrison et al., 2024). Moreover, the PENELOPE-B trial, although recruiting patients at higher risk than PALLAS patients, also failed to show greater benefits for high-risk patients (Loibl et al., 2021). Notably, patients in the PENELOPE-B trial received only 1 year of palbociclib adjuvant therapy, and longer treatment durations might yield different results. The risk profile of the NATALEE trial was slightly lower than that of the MONARCH-E trial, with subgroup analyses of NATALEE trial data revealing benefits for both high- and low-risk patients in terms of IDFS (Slamon et al., 2024). In the subgroup analysis of this study, no significant differences in benefits were observed between the high- and low-risk groups, such as those based on stage and lymph node status. Therefore, the varying risk profiles of participants across the four studies may not be the primary reason for the disparate outcomes. Second, three different drugs were used as CDK4/6 inhibitors across the four RCTs: palbociclib, abemaciclib, and ribociclib. Notably, two studies involving palbociclib did not show benefits, whereas studies involving abemaciclib and ribociclib demonstrated greater benefits. Despite their similar mechanisms of action, these CDK4/6 inhibitors exhibit variable drug activities (Infante et al., 2016; Klein et al., 2018). Some preclinical data have suggested that abemaciclib exhibits greater lipophilicity, enabling quicker penetration into breast and brain tissues, with a greater endocrine therapy response rate than palbociclib (Braal et al., 2021; Infante et al., 2016; Malorni et al., 2018). Ribociclib has a high absorption rate than palbociclib (Braal et al., 2021). Additionally, varying administration methods and divergent side effects also significantly impact patient adherence (Zhu and Zhu, 2023; Groenland et al., 2020). Compliance in the PALLAS trial was relatively poor, with 42% of patients discontinuing treatment before the planned 2-year endpoint of palbociclib, a proportion substantially lower (18%) in the MONARCH-E trial. Hence, we posit that differential pharmacokinetics are the primary drivers of the disparities observed across the conclusions of the four RCTs, necessitating an extended follow-up period for further outcome observation. It is noteworthy that if longer follow-up studies continue to show no benefit of palbociclib in HR+, HER2- early breast cancer, it may not be applicable and could potentially lead to more adverse effects for these patients.

In this meta-analysis, we also assessed the incidence of toxicity associated with CDK4/6 inhibitors in adjuvant therapy. Our study revealed a significant association between CDK4/6 inhibitors and increased rates of adverse events (AEs), particularly a heightened risk of adverse hematological reactions, which was consistent with previous research findings (Thill and Schmidt, 2018; Desnoyers et al., 2020; Martel et al., 2018). Therefore, during the administration of CDK4/6 inhibitors to patients, enhanced routine blood monitoring should be implemented.

Neoadjuvant endocrine therapy has traditionally been limited to a minority of patients; nevertheless, interest in the activity of CDK4/6 inhibitors in early breast cancer prompted the initiation of four RCTs. We conducted a meta-analysis of these four RCTs, and the final results indicated that CDK4/6 inhibitors promote CCCA, which was defined as Ki67 ≤ 2.7% (Suman et al., 2022). Ki67 serves as a prognostic factor for breast cancer patients and can be utilized as a dynamic monitoring indicator for the efficacy of neoadjuvant therapy (Hurvitz et al., 2020; Cottu et al., 2018). This discovery aligns with previous research findings (Guan et al., 2023). Additionally, a single-arm trial also revealed significant benefits of palbociclib combined with endocrine therapy in the neoadjuvant treatment of HR-positive/HER2-negative early breast cancer (Ma et al., 2017). These findings collectively indicate the feasibility of CDK4/6 inhibitors in neoadjuvant therapy for breast cancer. Notably, two studies (Alsaleh et al., 2023; Johnston et al., 2019) reported PCR data and demonstrated no benefit of CDK4/6 inhibitors in PCR. Therefore, further clinical data are required to explore the efficacy of CDK4/6 inhibitors in neoadjuvant therapy.

This study has several limitations. First, the number of studies we could include was limited, which prevented some sensitivity analyses and may also be a primary source of heterogeneity. Second, the patient recruitment criteria varied significantly among studies, and different studies utilized various CDK4/6 inhibitors (palbociclib, abemaciclib, and ribociclib) and treatment durations (1, 2, and 3 years), potentially restricting the interpretability of the pooled results. Third, most studies had short follow-up durations. As of the writing of this manuscript, only 20% of patients in the NATALEE study had completed a 3-year treatment; thus, the survival data remain immature. Last, due to limited data on neoadjuvant therapy, the incidence rates of AEs could not be analyzed. Despite these limitations, this meta-analysis represents the most comprehensive and up-to-date assessment of the role of CDK4/6 inhibitors in adjuvant and neoadjuvant therapy for early breast cancer. In addition, compared to previous meta-analyses, our study incorporated a recently published NATALEE study and observed a notable improvement in IDFS associated with CDK4/6 inhibitors in the treatment of early-stage breast cancer, but previous meta-analyses failed to achieve statistical significance. At last, In neoadjuvant trials, only two studies (SAFIA and PALLET) set PCR as an endpoint. Notably, both of these two studies used palbociclib in the experimental group, indicating that we cannot determine whether Ribociclib or Abemaciclib benefits from PCR.

In conclusion, this study revealed that CDK4/6 inhibitors can enhance the efficacy of adjuvant and neoadjuvant therapy in HR+/HER2- early breast cancer patients. Furthermore, we observed that the diversity of CDK4/6 inhibitors may be a major contributing factor to the inconsistency of previous research findings. These findings will further augment interest in the research of CDK4/6 inhibitors. However, additional clinical research data and longer follow-up results are needed for validation of these findings.

## Data Availability

The original contributions presented in the study are included in the article/supplementary material, further inquiries can be directed to the corresponding author.

## References

[B1] Agostinetto E. Vian L. Caparica R. Bruzzone M. Ceppi M. Lambertini M. (2021). CDK4/6 inhibitors as adjuvant treatment for hormone receptor-positive, HER2-negative early breast cancer: a systematic review and meta-analysis. ESMO Open 6 (2), 100091. 10.1016/j.esmoop.2021.100091 33743330 PMC8010395

[B2] Alsaleh K. Al Zahwahry H. Bounedjar A. Oukkal M. Saadeddine A. Mahfouf H. (2023). Neoadjuvant endocrine therapy with or without palbociclib in low-risk patients: a phase III randomized double-blind SAFIA trial. J. Cancer Res. Clin. Oncol. 149 (9), 6171–6179. 10.1007/s00432-023-04588-3 36680581 PMC9864499

[B3] Braal C. L. Jongbloed E. M. Wilting S. M. Mathijssen R. H. J. Koolen S. L. W. Jager A. (2021). Inhibiting CDK4/6 in breast cancer with palbociclib, ribociclib, and abemaciclib: similarities and differences. Drugs 81 (3), 317–331. 10.1007/s40265-020-01461-2 33369721 PMC7952354

[B4] Burstein H. J. (2020). Systemic therapy for estrogen receptor-positive, HER2-negative breast cancer. N. Engl. J. Med. 383 (26), 2557–2570. 10.1056/NEJMra1307118 33369357

[B5] Burstein H. J. Curigliano G. Thürlimann B. Weber W. P. Poortmans P. Regan M. M. (2021). Customizing local and systemic therapies for women with early breast cancer: the St. Gallen International Consensus Guidelines for treatment of early breast cancer 2021. Ann. Oncol. 32 (10), 1216–1235. 10.1016/j.annonc.2021.06.023 34242744 PMC9906308

[B6] Cardoso F. Spence D. Mertz S. Corneliussen-James D. Sabelko K. Gralow J. (2018). Global analysis of advanced/metastatic breast cancer: decade report (2005-2015). Breast 39, 131–138. 10.1016/j.breast.2018.03.002 29679849

[B7] Cottu P. D'Hondt V. Dureau S. Lerebours F. Desmoulins I. Heudel P. E. (2018). Letrozole and palbociclib versus chemotherapy as neoadjuvant therapy of high-risk luminal breast cancer. Ann. Oncol. 29 (12), 2334–2340. 10.1093/annonc/mdy448 30307466

[B8] Desnoyers A. Nadler M. B. Kumar V. Saleh R. Amir E. (2020). Comparison of treatment-related adverse events of different Cyclin-dependent kinase 4/6 inhibitors in metastatic breast cancer: a network meta-analysis. Cancer Treat. Rev. 90, 102086. 10.1016/j.ctrv.2020.102086 32861975

[B9] Egger M. Davey Smith G. Schneider M. Minder C. (1997). Bias in meta-analysis detected by a simple, graphical test. Bmj 315 (7109), 629–634. 10.1136/bmj.315.7109.629 9310563 PMC2127453

[B10] Eggersmann T. K. Degenhardt T. Gluz O. Wuerstlein R. Harbeck N. (2019). CDK4/6 inhibitors expand the therapeutic options in breast cancer: palbociclib, ribociclib and abemaciclib. BioDrugs 33 (2), 125–135. 10.1007/s40259-019-00337-6 30847853

[B11] Giuliano M. Schettini F. Rognoni C. Milani M. Jerusalem G. Bachelot T. (2019). Endocrine treatment versus chemotherapy in postmenopausal women with hormone receptor-positive, HER2-negative, metastatic breast cancer: a systematic review and network meta-analysis. Lancet Oncol. 20 (10), 1360–1369. 10.1016/s1470-2045(19)30420-6 31494037

[B12] Gnant M. Dueck A. C. Frantal S. Martin M. Burstein H. J. Greil R. (2022). Adjuvant palbociclib for early breast cancer: the PALLAS trial results (ABCSG-42/AFT-05/BIG-14-03). J. Clin. Oncol. 40 (3), 282–293. 10.1200/jco.21.02554 34874182 PMC10476784

[B13] Goetz M. P. Toi M. Campone M. Sohn J. Paluch-Shimon S. Huober J. (2017). MONARCH 3: abemaciclib as initial therapy for advanced breast cancer. J. Clin. Oncol. 35 (32), 3638–3646. 10.1200/jco.2017.75.6155 28968163

[B14] Gomes I. Abreu C. Costa L. Casimiro S. (2023). The evolving pathways of the efficacy of and resistance to CDK4/6 inhibitors in breast cancer. Cancers (Basel) 15 (19), 4835. 10.3390/cancers15194835 37835528 PMC10571967

[B15] Groenland S. L. Martínez-Chávez A. van Dongen M. G. J. Beijnen J. H. Schinkel A. H. Huitema A. D. R. (2020). Clinical pharmacokinetics and pharmacodynamics of the cyclin-dependent kinase 4 and 6 inhibitors palbociclib, ribociclib, and abemaciclib. Clin. Pharmacokinet. 59 (12), 1501–1520. 10.1007/s40262-020-00930-x 33029704

[B16] Guan Y. Shen G. Fang Q. Xin Y. Huo X. Li J. (2023). Cyclin-dependent kinase 4 and 6 inhibitors in combination with neoadjuvant endocrine therapy in estrogen receptor-positive early breast cancer: a systematic review and meta-analysis. Clin. Exp. Med. 23 (2), 245–254. 10.1007/s10238-022-00814-3 35304677

[B17] Harbeck N. Gnant M. (2017). Breast cancer. Lancet 389 (10074), 1134–1150. 10.1016/s0140-6736(16)31891-8 27865536

[B18] Haslam A. Ranganathan S. Prasad V. Olivier T. (2024). CDK4/6 inhibitors as adjuvant therapy in early breast cancer? Uncertain benefits, guaranteed harms. Eur. J. Cancer 207, 114192. 10.1016/j.ejca.2024.114192 38959677

[B19] Higgins J. P. Altman D. G. Gøtzsche P. C. Jüni P. Moher D. Oxman A. D. (2011). The Cochrane Collaboration's tool for assessing risk of bias in randomised trials. Bmj 343, d5928. 10.1136/bmj.d5928 22008217 PMC3196245

[B20] Higgins J. P. Thompson S. G. Deeks J. J. Altman D. G. (2003). Measuring inconsistency in meta-analyses. Bmj 327 (7414), 557–560. 10.1136/bmj.327.7414.557 12958120 PMC192859

[B21] Hurvitz S. A. Martin M. Press M. F. Chan D. Fernandez-Abad M. Petru E. (2020). Potent cell-cycle inhibition and upregulation of immune response with abemaciclib and anastrozole in neoMONARCH, phase II neoadjuvant study in HR(+)/HER2(-) breast cancer. Clin. Cancer Res. 26 (3), 566–580. 10.1158/1078-0432.Ccr-19-1425 31615937 PMC7498177

[B22] Infante J. R. Cassier P. A. Gerecitano J. F. Witteveen P. O. Chugh R. Ribrag V. (2016). A phase I study of the cyclin-dependent kinase 4/6 inhibitor ribociclib (LEE011) in patients with advanced solid tumors and lymphomas. Clin. Cancer Res. 22 (23), 5696–5705. 10.1158/1078-0432.Ccr-16-1248 27542767 PMC5621377

[B23] Johnston S. Puhalla S. Wheatley D. Ring A. Barry P. Holcombe C. (2019). Randomized phase II study evaluating palbociclib in addition to letrozole as neoadjuvant therapy in estrogen receptor-positive early breast cancer: PALLET trial. J. Clin. Oncol. 37 (3), 178–189. 10.1200/jco.18.01624 30523750

[B24] Johnston S. R. D. Harbeck N. Hegg R. Toi M. Martin M. Shao Z. M. (2020). Abemaciclib combined with endocrine therapy for the adjuvant treatment of HR+, HER2-node-positive, high-risk, early breast cancer (monarchE). J. Clin. Oncol. 38 (34), 3987–3998. 10.1200/jco.20.02514 32954927 PMC7768339

[B25] Johnston S. R. D. Toi M. O'Shaughnessy J. Rastogi P. Campone M. Neven P. (2023). Abemaciclib plus endocrine therapy for hormone receptor-positive, HER2-negative, node-positive, high-risk early breast cancer (monarchE): results from a preplanned interim analysis of a randomised, open-label, phase 3 trial. Lancet Oncol. 24 (1), 77–90. 10.1016/s1470-2045(22)00694-5 36493792 PMC11200328

[B26] Kalinsky K. Accordino M. K. Chiuzan C. Mundi P. S. Sakach E. Sathe C. (2023). Randomized phase II trial of endocrine therapy with or without ribociclib after progression on cyclin-dependent kinase 4/6 inhibition in hormone receptor-positive, human epidermal growth factor receptor 2-negative metastatic breast cancer: MAINTAIN trial. J. Clin. Oncol. 41 (24), 4004–4013. 10.1200/jco.22.02392 37207300

[B27] Khan Q. O'dea A. Bardia A. Kalinsky K. Wisinski K. O'regan R. (2020). Letrozole + ribociclib versus letrozole + placebo as neoadjuvant therapy for ER+ breast cancer (FELINE trial). J. Clin. Oncol. 38, 505. 10.1200/jco.2020.38.15_suppl.505

[B28] Klein M. E. Kovatcheva M. Davis L. E. Tap W. D. Koff A. (2018). CDK4/6 inhibitors: the mechanism of action may not Be as simple as once thought. Cancer Cell 34 (1), 9–20. 10.1016/j.ccell.2018.03.023 29731395 PMC6039233

[B29] Li J. Fu F. Yu L. Huang M. Lin Y. Mei Q. (2020b). Cyclin-dependent kinase 4 and 6 inhibitors in hormone receptor-positive, human epidermal growth factor receptor-2 negative advanced breast cancer: a meta-analysis of randomized clinical trials. Breast Cancer Res. Treat. 180 (1), 21–32. 10.1007/s10549-020-05528-2 31970560

[B30] Li J. Huo X. Zhao F. Ren D. Ahmad R. Yuan X. (2020a). Association of cyclin-dependent kinases 4 and 6 inhibitors with survival in patients with hormone receptor-positive metastatic breast cancer: a systematic review and meta-analysis. JAMA Netw. Open 3 (10), e2020312. 10.1001/jamanetworkopen.2020.20312 33048129 PMC8094425

[B31] Loibl S. Marmé F. Martin M. Untch M. Bonnefoi H. Kim S. B. (2021). Palbociclib for residual high-risk invasive HR-positive and HER2-negative early breast cancer-the Penelope-B trial. J. Clin. Oncol. 39 (14), 1518–1530. 10.1200/jco.20.03639 33793299

[B32] Lu Y. S. Im S. A. Colleoni M. Franke F. Bardia A. Cardoso F. (2022). Updated overall survival of ribociclib plus endocrine therapy versus endocrine therapy alone in pre- and perimenopausal patients with HR+/HER2- advanced breast cancer in MONALEESA-7: a phase III randomized clinical trial. Clin. Cancer Res. 28 (5), 851–859. 10.1158/1078-0432.Ccr-21-3032 34965945 PMC9377723

[B33] Ma C. X. Gao F. Luo J. Northfelt D. W. Goetz M. Forero A. (2017). NeoPalAna: neoadjuvant palbociclib, a cyclin-dependent kinase 4/6 inhibitor, and anastrozole for clinical stage 2 or 3 estrogen receptor-positive breast cancer. Clin. Cancer Res. 23 (15), 4055–4065. 10.1158/1078-0432.Ccr-16-3206 28270497 PMC5555232

[B34] Malorni L. Curigliano G. Minisini A. M. Cinieri S. Tondini C. A. D'Hollander K. (2018). Palbociclib as single agent or in combination with the endocrine therapy received before disease progression for estrogen receptor-positive, HER2-negative metastatic breast cancer: TREnd trial. Ann. Oncol. 29 (8), 1748–1754. 10.1093/annonc/mdy214 29893790 PMC6454527

[B35] Martel S. Bruzzone M. Ceppi M. Maurer C. Ponde N. F. Ferreira A. R. (2018). Risk of adverse events with the addition of targeted agents to endocrine therapy in patients with hormone receptor-positive metastatic breast cancer: a systematic review and meta-analysis. Cancer Treat. Rev. 62, 123–132. 10.1016/j.ctrv.2017.09.009 29108713

[B36] Moher D. Liberati A. Tetzlaff J. Altman D. G. PRISMA Group (2009). Preferred reporting items for systematic reviews and meta-analyses: the PRISMA statement. Bmj 339, b2535. 10.1136/bmj.b2535 19622551 PMC2714657

[B37] Morrison L. Loibl S. Turner N. C. (2024). The CDK4/6 inhibitor revolution - a game-changing era for breast cancer treatment. Nat. Rev. Clin. Oncol. 21 (2), 89–105. 10.1038/s41571-023-00840-4 38082107

[B38] O’Sullivan C. C. Clarke R. Goetz M. P. Robertson J. (2023). Cyclin-dependent kinase 4/6 inhibitors for treatment of hormone receptor-positive, ERBB2-negative breast cancer: a review. JAMA Oncol. 9 (9), 1273–1282. 10.1001/jamaoncol.2023.2000 37382948 PMC11385778

[B39] Piezzo M. Cocco S. Caputo R. Cianniello D. Gioia G. D. Lauro V. D. (2020). Targeting cell cycle in breast cancer: CDK4/6 inhibitors. Int. J. Mol. Sci. 21 (18), 6479. 10.3390/ijms21186479 32899866 PMC7554788

[B40] Rastogi P. O'Shaughnessy J. Martin M. Boyle F. Cortes J. Rugo H. S. (2024). Adjuvant abemaciclib plus endocrine therapy for hormone receptor-positive, human epidermal growth factor receptor 2-negative, high-risk early breast cancer: results from a preplanned monarchE overall survival interim analysis, including 5-year efficacy outcomes. J. Clin. Oncol. 42 (9), 987–993. 10.1200/jco.23.01994 38194616 PMC10950161

[B41] Slamon D. Lipatov O. Nowecki Z. McAndrew N. Kukielka-Budny B. Stroyakovskiy D. (2024). Ribociclib plus endocrine therapy in early breast cancer. N. Engl. J. Med. 390 (12), 1080–1091. 10.1056/NEJMoa2305488 38507751

[B42] Suman V. J. Du L. Hoskin T. Anurag M. Ma C. Bedrosian I. (2022). Evaluation of sensitivity to endocrine therapy index (SET2,3) for response to neoadjuvant endocrine therapy and longer-term breast cancer patient outcomes (alliance Z1031). Clin. Cancer Res. 28 (15), 3287–3295. 10.1158/1078-0432.Ccr-22-0068 35653124 PMC9357183

[B43] Thill M. Schmidt M. (2018). Management of adverse events during cyclin-dependent kinase 4/6 (CDK4/6) inhibitor-based treatment in breast cancer. Ther. Adv. Med. Oncol. 10, 1758835918793326. 10.1177/1758835918793326 30202447 PMC6122233

[B44] Zhang X. Zhou Q. Qi Y. Chen X. Deng J. Zhang Y. (2024). The effect of tomato and lycopene on clinical characteristics and molecular markers of UV-induced skin deterioration: a systematic review and meta-analysis of intervention trials. Crit. Rev. Food Sci. Nutr. 64 (18), 6198–6217. 10.1080/10408398.2022.2164557 36606553

[B45] Zhu Z. Zhu Q. (2023). Differences in metabolic transport and resistance mechanisms of Abemaciclib, Palbociclib, and Ribociclib. Front. Pharmacol. 14, 1212986. 10.3389/fphar.2023.1212986 37475713 PMC10354263

