# Supplementary figures and images for: Adjuvant and neoadjuvant therapy with or without CDK4/6 inhibitors in HR+/HER2- early breast cancer: a systematic review and meta-analysis
Source: Front Pharmacol. 2024 Sep 12;15:1438288. doi: 10.3389/fphar.2024.1438288 (PMC11424878; doi:10.3389/fphar.2024.1438288)

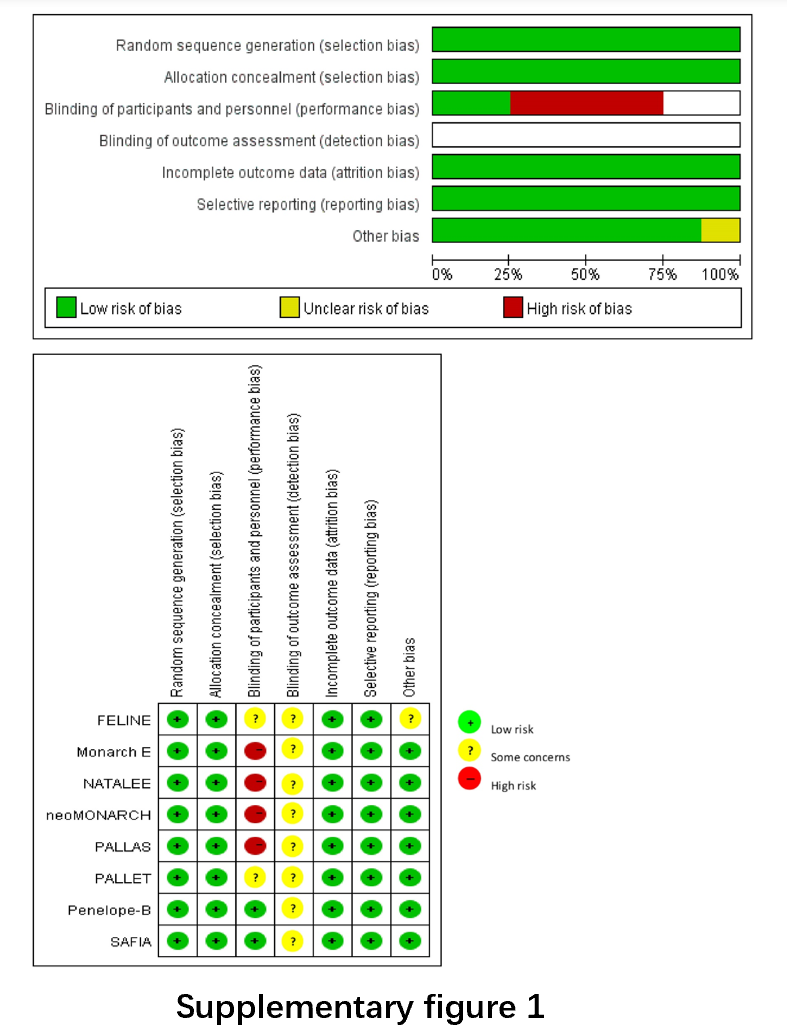

Supplement: Supplementary file 1 [file Image1.PNG]
