# Supplementary material for: Adjuvant and neoadjuvant therapy with or without CDK4/6 inhibitors in HR+/HER2- early breast cancer: a systematic review and meta-analysis
Source: Front Pharmacol. 2024 Sep 12;15:1438288. doi: 10.3389/fphar.2024.1438288 (PMC11424878; doi:10.3389/fphar.2024.1438288)
Supplement: Supplementary file 2 [file DataSheet1.DOCX]

| Database | Search terms |
| --- | --- |
| PubMed | 1. ((palbociclib[Title/Abstract]) OR (ribociclib[Title/Abstract])) OR (abemaciclib[Title/Abstract]) 2. (((((cyclin-dependent kinase 4[Title/Abstract] AND 6 inhibitor*[Title/Abstract]) OR (cyclin-dependent kinase 4/6 inhibitor*[Title/Abstract])) OR (CDK4[Title/Abstract] AND CDK6 inhibitor*[Title/Abstract])) OR (CDK4/6 inhibitor*[Title/Abstract])) OR (CDK inhibitor*[Title/Abstract])) OR (CDKi[Title/Abstract]) 3. #1 OR #2 4. (((((((Breast Neoplasm*[Title/Abstract]) OR (Breast Tumor*[Title/Abstract])) OR (Breast Cancer*[Title/Abstract])) OR (Breast Carcinoma*[Title/Abstract])) OR (Mammary Neoplasm*[Title/Abstract])) OR (Mammary Tumor*[Title/Abstract])) OR (Mammary Cancer*[Title/Abstract])) OR (Mammary Carcinoma*[Title/Abstract]) 5. #3 AND #4 |
| Embase | 1. ((cyclin-dependent kinase 4 and 6 inhibitor*) or (CDK4 and CDK6 inhibitor*) or cyclin-dependent kinase 4 6 inhibitor* or CDK4 6 inhibitor* or CDK inhibitor* or CDKi). ti,ab,tw. 2. (palbociclib or ribociclib or abemaciclib). ti,ab,tw. 3. 1 OR 2 4. (Breast Neoplasm* or Breast Tumor* or Breast Cancer* or Breast Carcinoma* or Mammary Neoplasm* or Mammary Tumor* or Mammary Cancer* or Mammary Carcinoma*). ti,ab,tw. 5. 3 AND 4 |

Supplementary Table 1: The search strategies for different databases.

Supplementary Table 2: Recruitment criteria for included adjuvant trails.

|  | NATALEE | | PALLAS | | MonarchE | | PENELOPE-B | |
| --- | --- | --- | --- | --- | --- | --- | --- | --- |
|  | ribociclib + ET | ET alone | ribociclib + ET | ET alone | ribociclib + ET | ET alone | ribociclib + ET | ET alone |
| N | 2549 | 2552 | 2883 | 2877 | 2808 | 2829 | 631 | 619 |
| Median follow-up (months) | 30 |  | 23.7 |  | 19.1 |  | 42.8 |  |
| CDK4/6i duration (years) | 3 |  | 2 |  | 2 |  | 1 |  |
| Stage, n (%) |  |  |  |  |  |  |  |  |
| I | 9 (0.4) | 5 (0.2) | - | - | 2(0.1) | 1(0.1) | - | - |
| IIA | 479 (18.8) | 521 (20.4) | 504 (17.5) | 509 (17.7) | 323 (11.5) | 353 (12.5) | - | - |
| IIB | 532 (20.9) | 513 (20.1) | 968 (33.6) | 951 (33.1) | 389 (13.9) | 387 (13.7) | - | - |
| III | 1528 (59.9) | 1512 (59.2) | 1402 (48.6) | 1408 (48.9) | 2081 (74.1) | 2077 (73.4) | - | - |
| Tumor size, n (%) |  |  |  |  |  |  |  |  |
| T1-2 | - | - | 2160 (74.9) | 2136 (74.1) | 2149 (76.6) | 2184 (77.2) | 606 (96) | 597 (96.6) |
| T3-4 | - | - | 722 (25.0) | 741 (25.8) | 610 (21.7) | 612 (21.6) | 25 (4) | 21 (3.4) |
| Grade, n (%) |  |  |  |  |  |  |  |  |
| G1 | 218 (8.6) | 240 (9.4) | 300 (10.4) | 313 (10.9) | 209 (7.4) | - | - |  |
| G2 | 1458 (57.2) | 1451 (56.9) | 1622 (56.3) | 1658 (57.6) | 1373 (48.9) | - | - |  |
| G3 | 521 (20.4) | 549 (21.5) | 836 (29.0) | 767 (26.7) | 1090 (38.8) | 294 (46.7) | 297 (48.1) |  |
| Node status |  |  |  |  |  |  |  |  |
| N0-1 | 1440 (56.5) | 1457 (57.1) | 1794 (62.2) | 1798 (62.5) | - | - | 310 (49.1) | 310(50.1) |
| N2-3 | 1105 (43.4) | 1089 (42.7) | 1088 (37.8) | 1079 (37.5) | - | - | 321 (50.9) | 309 (49.9) |
| Ovarian suppression (any time), n (%) | 670 (26.3) | 620 (24.3) | 532 (18.5) | 604 (21.1) | 606 (21.7) | 627 (22.4) | 108 (17.1) | 113 (18.3) |
| Early CDK4/6i discontinuation, n (%) | 477 (18.9%) |  | 772 (26.7) |  | 481 (17.2) |  | 33 (5.2) |  |

ET, endocrine therapy

**Supplementary Table 3: Leave-one-out sensitivity analysis for invasive disease-free survival.**

| **Study excluded** | **HR (95% CI)** | **P** | **I^2^** | **Model** |
| --- | --- | --- | --- | --- |
| NATALEE | 0.84 (0.66, 1.07) | 0.162 | 84% | Random |
| PALLAS | 0.76 (0.64, 0.91) | 0.002 | 61.4% | Random |
| MonarchE | 0.88 (0.78, 0.98) | 0.021 | 48.5% | Fixed |
| Penelope-B | 0.79 (0.64, 0.97) | 0.024 | 80.5% | Random |

CI, confidence interval; HR, hazard ratio.

**Supplementary Table 4: Leave-one-out sensitivity analysis for CCCA.**

| **Study excluded** | **RR (95% CI)** | **P** | **I^2^** | **Model** |
| --- | --- | --- | --- | --- |
| PALLET | 2.74 (1.03, 7.32) | 0.044 | 86.1% | Random |
| neoMonarchE | 1.61 (1.35, 1.91) | <0.001 | 0% | Fixed |
| FELINE | 2.59 (0.87, 7.73) | 0.089 | 89.9% | Random |

RR, risk ratio; CCCA, complete cell-cycle arrest
